# Fifty Years of Supporting Global Health Research at the NIH Fogarty International Center

**DOI:** 10.5334/aogh.2432

**Published:** 2019-03-20

**Authors:** Kenneth Bridbord, Kristen H. Weymouth, Ann Puderbaugh, Celia Wolfman, Christopher W. Belter, Joel G. Breman, Peter H. Kilmarx

**Affiliations:** 1 National Institutes of Health (NIH), Fogarty International Center, Bethesda, Maryland, US; 2 National Institutes of Health (Library), Office of Research Services, Bethesda, Maryland, US

## Abstract

For 50 years, the Fogarty International Center (FIC) has built research capacity particularly in low and middle-income countries responding to national and global public health priorities. Established in 1968 in honor of U.S. Congressman John E. Fogarty, FIC is one of 27 Institutes and Centers at the U.S. National Institutes of Health (NIH). Initially created in response to the HIV/AIDS pandemic in the 1980s and emerging infectious diseases in the 1990s, the Center provided training for approximately 6,000 health scientists from more than 100 countries including 1,000 from the U.S. Current programs are catalytic, addressing national and international institutional capacity strengthening in HIV and other infectious diseases, environmental and occupational health, research ethics, brain disorders, trauma and injury and other non-communicable diseases, tobacco, health systems implementation research, and medical education. Since 1988, FIC provided over $1.5 billion in extramural grants leveraging its relatively modest $50 million extramural budget by $20–$30 million annually. FIC-trained scientists and public health leaders led key studies about malaria vaccines and AIDS prevention trials, became directors of national HIV/AIDS programs, and achieved leadership positions such as Minister of Health. Between 2009 and 2015, FIC cited-papers averaged approximately 1.1% of the NIH total, in comparison to the FIC budget, which averaged only 0.22% of the NIH budget. While maintaining strong commitments to respond to global health threats caused by communicable diseases, FIC is training the next generation of global health researchers focusing on chronic diseases, implementation science and epidemic modeling needed to predict and help contain future global pandemics.

## History

In 2018, the Fogarty International Center (FIC) marked its 50^th^ anniversary at the U.S. National Institutes of Health (NIH). FIC is named in honor of the late John E. Fogarty, a long-term Congressman from Rhode Island and champion of NIH. Under Congressman Fogarty, who was chairman of the Appropriations Subcommittee that determines NIH funding, the agency experienced one of its greatest periods of growth—from a budget of $37 million in 1949, to $1.24 billion in 1967.

Since its formation, FIC has served as a bridge between NIH and the global health community, facilitating exchanges among investigators, providing training opportunities and supporting promising research initiatives in developing countries. FIC programs have provided research training for about 6,000 health scientists worldwide from more than 100 nations, including about 1,000 from the U.S. Many of these individuals have become national scientific and global health leaders.

FIC’s programs have made pivotal contributions filling the pipeline of global health leaders in low and middle-income countries (LMICs), and the U.S., extending the frontiers of science and accelerating discovery. FIC has invested and will continue to invest in people—the most important resource in global health research—serving on the front lines of the fight against diseases that threaten populations worldwide.

Congressman Fogarty dreamt of establishing an institution where the best scientists from around the world would collaborate to solve the most challenging global health problems. Though the Congressman never lived to see his dream come true due to his sudden death in 1967 at age 53, his “Health for Peace” Center became a reality when his colleagues in Congress passed a resolution to establish the Fogarty Center at NIH in his honor. On July 1, 1968, President Lyndon Johnson issued an Executive Order establishing the John E. Fogarty International Center for Advanced Study in the Health Sciences at NIH.

The current mission statement reads, “The Fogarty International Center is dedicated to advancing the mission of the National Institutes of Health (NIH) by supporting and facilitating global health research conducted by U.S. and international investigators, building partnerships between health research institutions in the U.S. and abroad, and training the next generation of scientists to address global health needs.”

For FIC, this mission translates into advancing science and strengthening research and public health capacity in areas where resources are limited and challenges to health involve a dual burden of communicable and non-communicable diseases (NCDs). FIC focuses on LMICs that face enormous disease burdens but lack scientific capacity to study these problems or adapt established interventions to suit their context and resources. FIC investments in human resources accelerated the progress of research, and the scientific enterprise building research capacity at institutions across the globe, and, most importantly, developing the knowledge and evidence needed to confront health challenges locally and globally.

As LMICs’ research programs matured, benefits also became increasingly bidirectional. For instance, FIC trainees provide local capacity to detect and address disease outbreaks where they occur, helping contain pandemics and contributing to global health security. FIC trainees capitalize on unique research opportunities, for example, studying the Zika virus in Brazil and Peru, or testing a drug that may prevent Alzheimer’s disease among a family in Colombia with an inherited early-onset form of the disease [1].

## Key Achievements

FIC’s most important contributions during its first two decades were a series of international conferences that stimulated the global campaign to eliminate polio and expand childhood immunizations, and supporting scholars’ exchanges, mainly with high-income countries [2,3]. Today FIC is best known for building research capacity and scientific leadership in LMICs. This began with creation of the AIDS International Training and Research Program (AITRP) in 1988. AITRP and its successor programs have been a powerful force building sustainable research capacity at many institutions in LMICs focused on HIV/AIDS and developing long-standing partnerships between U.S. and LMIC institutions (Table 1). This capacity is reflected by the success of these institutions competing for research and research training awards from NIH and other funders. Individuals trained through these programs have become national and global health leaders (Table 2).

**Table 1:** Sustainable Research Capacity in Select LMICs Facilitated by Partnerships Under FIC Research and Research Training Programs.

Foreign Institution	U.S. Institutions

The International Centre for Diarrhoeal Disease Research, Bangladesh (icddr, b)	University of Chicago, Johns Hopkins University (JHU), Massachusetts General Hospital
University of Botswana	Harvard University, Baylor University
Chinese Center for Disease Control and Prevention (CDC)	University of California, Los Angeles (UCLA)
Addis Adaba University, Ethiopia	Emory University, JHU
The Haitian Group for the Study of Kaposi’s Sarcoma and Opportunistic Infections (GHESKIO), Haiti	Cornell University
University of Nairobi, Kenya	University of Washington, University of Maryland–Baltimore (UMBC)
Kenya Medical Research Institute (KEMRI)	University of Washington, University of California Berkeley (UCB)
University of Malawi	University of North Carolina (UNC), JHU
University of Ibadan, Nigeria	Northwestern University, Harvard University, UMBC
Cayetano Heredia, Peru	University of Washington, JHU, UCB
Muhimbili University of Health and Allied Sciences (MUHAS), Tanzania	Harvard University, Dartmouth College
University of KwaZulu-Natal, South Africa	Columbia University
University of Cape Town, South Africa	JHU
Kilimanjaro Christian Medical Centre (KCMC), Tanzania	Duke University
Makerere University, Uganda	JHU, Case Western Reserve University, UCB/University of California San Francisco (UCSF)
Mbarra University, Uganda	University of Georgia
University of Zambia	University of Alabama/Vanderbilt University, UNC
University of Zimbabwe	Stanford University, University of Colorado-Denver

**Table 2:** Select, Notable LMIC Scientific Leaders Involved in Research Training Programs Supported by the Fogarty International Center.

Name and Country	Current Position

Jane Aceng Uganda	Minister of Health, Uganda
Isaac Folorunso Adewole Nigeria	Minister of Health, Nigeria
Daphne Benoit Haiti	Minister of Health, Haiti
Awa Coll-Seck Senegal	Minister of Health, Senegal; Executive Director, Roll Back Malaria Partnership
Marcos Espinal Dominican Republic	Chief, Department of Communicable Diseases and Health Analysis, WHO’s Pan American Health Organization (PAHO)
Linda Gail-Bekker South Africa	Deputy Director of the Desmond Tutu HIV Centre and President of the International AIDS Society (IAS)
Patricia Garcia Peru	Professor and Dean of the School of Public Health at Cayetano Heredia University, former Minister of Health, Peru, and Director of the Peruvian NIH
Glenda Gray South Africa	Current Head of the South Africa Medical Research Council (MRC) and Chair of the Global Alliance for Chronic Diseases (GACD)
Florance Guillaume Haiti	Minister of Health, Haiti
Quarraisha Abdool Karim South Africa	Director of the FIC AIDS Training Program in South Africa and Associate Scientific Director of Centre for the AIDS Programme of Research in South Africa
Salim Abdool Karim South Africa	Director of the Centre for the AIDS Programme of Research (CAPRISA), former Head of the Medical Research Council (MRC) in South Africa
Dan Namarika Malawi	Secretary of Health, Malawi
John Nkengasong Ethiopia	Director, Africa Centres for Disease Control and Prevention
Jean “Bill” Pape Haiti	Director, The Haitian Group for the Study of Kaposi’s Sarcoma and Opportunistic Infections (GHESKIO)
Adolfo Rubinstein Argentina	Minister of Health, Argentina
Nelson Sewankambo Uganda	Professor of Medicine and the Principal (Head) of Makerere University College of Health Sciences, former Dean of Makerere University Medical School
Suniti Solomon India (deceased)	Founder-director of Y.R. Gaitonde Center for AIDS Research and Education (YRGCARE)
Soumya Swaminathan India	Deputy Director General, WHO and former Director General, Indian Council of Medical Research (ICMR)
Elioda Tumwesigye Uganda	Minister of Health and Minister of Science, Technology and Innovation in Uganda
Zunyu Wu China	Director of the AIDS Program at the National Center for Disease Control (CDC) in China

Many scientific advances, such as male circumcision to reduce HIV transmission, prevention of mother-to-child HIV transmission (PMTCT) and antiretroviral (ARV) treatment as prevention were made by FIC trainees and AITRP collaborators [1,4].

FIC has contributed to advancing global health by building research capacity within and outside NIH, essential for advancement of biomedical research to develop new cures, treatments, knowledge and public health interventions. For nearly ten years, NIH has reported to the U.S. Congress on the number of its programs representing collaborations with one or more Institutes and Centers (ICs). Over these years, FIC has consistently ranked as the most collaborative across NIH at a rate 4–5 times the NIH average.

FIC subsequently developed cross-cutting research and research training programs designed to build a strong foundation in areas critical to conducting clinical research, including bioethics, informatics, implementation science, genetics, stigma and population studies. As the global disease burden shifted from infectious to chronic diseases, FIC responded with new programs supporting research and training on chronic NCDs, and initiatives studying how best to leverage HIV research and patient care delivery platforms to address NCDs.

One of the highest priorities for FIC is mentoring the next generation of global health scientists from both LMICs and the U.S. through the Global Health Fellows and Scholars Program which has provided more than 1,000 fellowships for both U.S. and foreign scientists [5]. Additional strategies include supporting institutional junior faculty development awards and providing more opportunities for LMIC investigators to compete for awards made directly to their institutions. Most recently, NIH has partnered with the Bill and Melinda Gates Foundation and the African Academy of Sciences to establish the African Postdoctoral Training Initiative. This program, coordinated by FIC, is designed to provide research opportunities for African scientists in NIH intramural laboratories with the goal of helping establish their careers at their home institutions.

Over the past 30 years, FIC has expanded into other areas for research and research training such as emerging infectious diseases, environmental/occupational health, trauma/injury and tobacco research. FIC has been a pioneer in implementation science (delivery) research and research training, initially through linking clinical, operational and health services research training in both HIV/AIDS and NCDs. FIC’s Division of International Training and Research now oversees more than two dozen programs and a total of about 500 extramural awards each year.

The need to explore new directions and expand efforts linking research to public health and health services practice was a major reason for establishing the Center for Global Health Studies (CGHS) within FIC. CGHS aims to identify emerging research priorities, stimulate new scientific directions in global health, and support multidisciplinary collaboration and short-term training addressing pressing global health problems. Among the major initial CGHS efforts were studies to better implement programs preventing mother-to-child HIV transmission and strategies expanding health services in HIV/AIDS to address NCDs associated with HIV infection [6,7].

FIC’s Division of International Epidemiology and Population Studies established a unique program, with support from other Federal agencies, to model and predict emergence of global health threats. Noteworthy studies predict the spread of influenza around the world using genetic epidemiology, focusing on disease patterns in northern and southern hemispheres [8]. FIC’s Division of International Relations plays a leadership role across NIH, tracking NIH international investments, initiatives, and partnerships, informing NIH and outside stakeholders; advising NIH Institutes, Centers and Offices (ICOs) of opportunities to enhance their global health portfolios; identifying potential partnerships with international governments, domestic, and international organizations; and providing guidance on country-specific laws and regulations potentially impacting international research.

During the last 30 years, and with substantial co-funding, FIC has invested over $1.5 billion to support research and research capacity building in LMICs. Notable is that since 2004, the FIC extramural core budget has averaged about $50 million yearly, with an additional $20–30 million coming from partners. Significant funding increases occurred in fiscal years (FY) 2010 and 2011 from the American Recovery and Reinvestment Act of 2009 and funds for the Medical Education Partnership Initiative (MEPI) program.

With funding from the President’s Emergency Program for AIDS Relief (PEPFAR) and NIH, MEPI was co-administered by FIC and the Health Resources and Services Administration (HRSA) [9]. MEPI has transformed medical education in Africa and led to a network of junior faculty training programs at African medical schools supported by MEPI. One early result of MEPI was formation of a continent-wide forum for health professional education in Africa (AFREhealth). MEPI made significant strides in strengthening the breadth and depth of curricula, advancing implementation of information communication technologies, and developing regional training centers providing health expertise where it is most lacking.

While over one hundred LMICs have benefitted from FIC programs, contributions to global health science have been particularly noteworthy in a number of selected countries, many with high HIV/AIDS prevalence. Contributions include:

- Use of isoniazid to prevent active tuberculosis (TB) and progression of HIV infection in persons infected by HIV and TB in Haiti [10]
- Effective antiretroviral therapy (ART) in increasing survival in persons with AIDS in Haiti [11]
- Improved retention and outcome in patients with same day HIV testing and treatment in Haiti [12]
- Documenting the cycle of HIV transmission in rural South Africa [13]
- Effectivene and safe use of Tenofovir vaginal gel in prevention of HIV infection in women in South Africa [14]
- Identification of broadly neutralizing antibodies against HIV in persons in South Africa [15]
- Strategies to better treat persons with HIV and TB co-infection in South Africa [16]
- Effectiveness studies of treatment for TB in Brazil [17]
- Studies of the impact of breastfeeding on HIV-1 infected women in Kenya [18]
- Studies of the impact of circumcision on STD transmission in Uganda [19]
- Studies of the effect of circumcision on HIV transmission to women in Uganda [20]
- Decreased heterosexual HIV transmission in Zambia and Rwanda [21]
- Prevention of mother-to-child HIV infection in Botswana [22]
- Prevention of HIV transmission in breastfeeding in Ethiopia, India and Uganda [23]
- Evolution of China’s response to HIV/AIDS [24]

**Figure 1:**
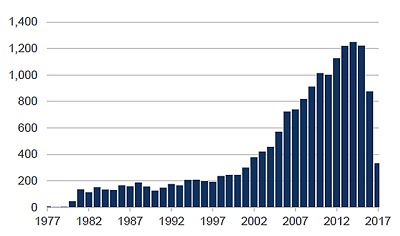
**Articles by year citing FIC support.** The decreases in publication output for 2016 and 2017 were due to indexing lags at the time this figure was generated, and not to decreases in FIC productivity.

One measure of the impact of FIC’s programs is illustrated by bibliometric analyses conducted by the NIH Library. Researchers identified over 15,000 articles citing FIC support beginning in 1978 through 2015 (Figure 1). The number of articles citing FIC rose steadily through the 1990s but increased dramatically from 2000, reflecting increased co-funding of FIC extramural programs beginning in 1993; expansion of FIC’s HIV research training programs beginning in 1998, and doubling of the NIH budget from 1998 to 2002.

**Figure 2:**
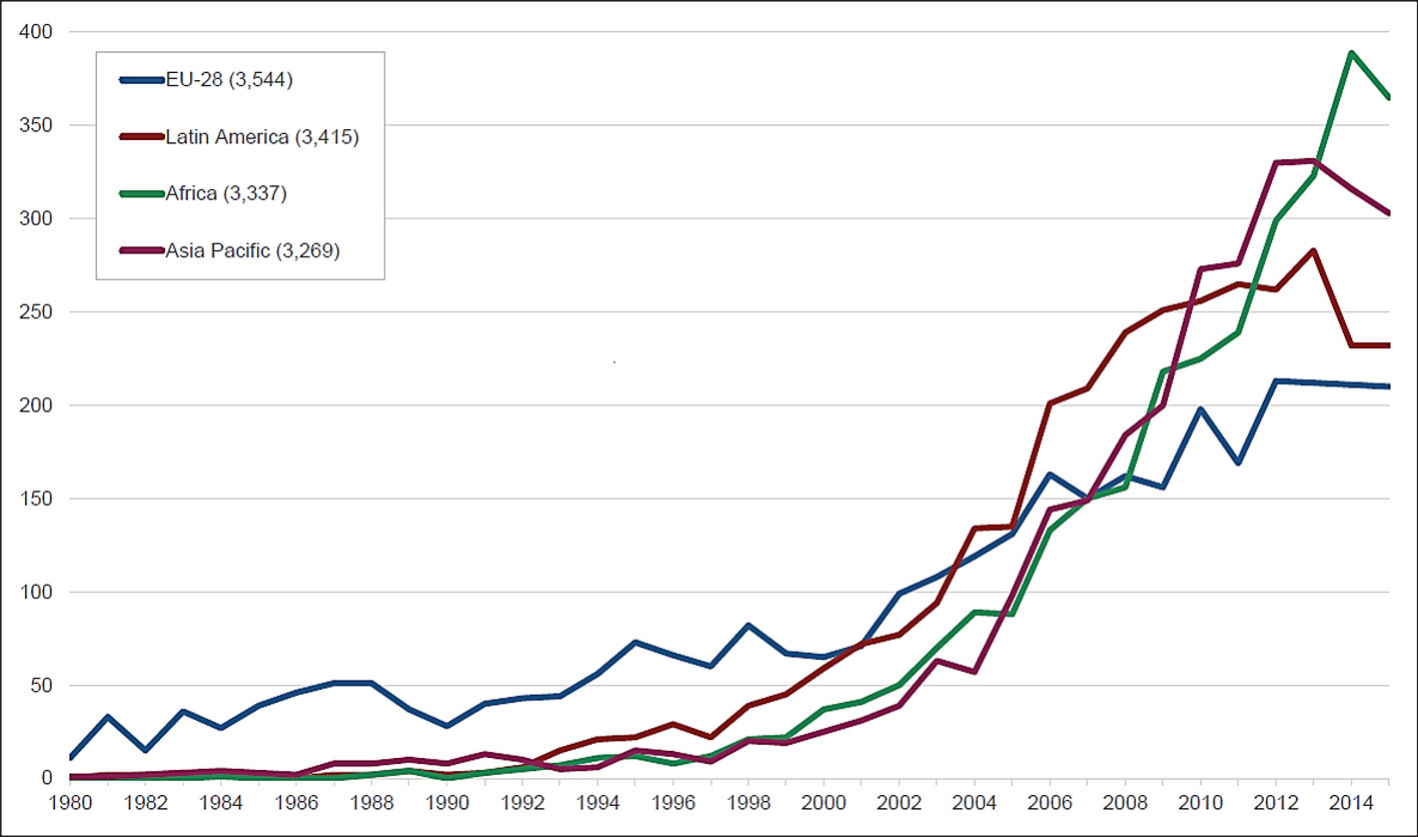
Articles with FIC support per author-affiliated region per year.

Papers citing FIC funding published during 2009–2015 averaged about 1,100 per year while total NIH-cited papers averaged ~100,000 per year (Figure 1). As a result, during this time period FIC-cited papers represented 1.1% of the NIH average. Given that FIC makes up ~0.22% of NIH’s budget, papers citing FIC funding were published at a rate five times the NIH average, a figure similar to an independent analysis of the sustained scientific productivity demonstrated by FIC-cited papers [1]. Programs supported by FIC have been credited with rebuilding biomedical research in Europe following WWII. Consequently, before 2000, the greatest foreign author affiliations were from European Union (EU) countries reflecting the historic focus of early FIC research and fellowship programs targeting that area of the world (Figure 2). Since 2001, the greatest foreign author affiliation has been with LMIC scientists, with Africa being the most cited region beginning in 2012.

**Figure 3:**
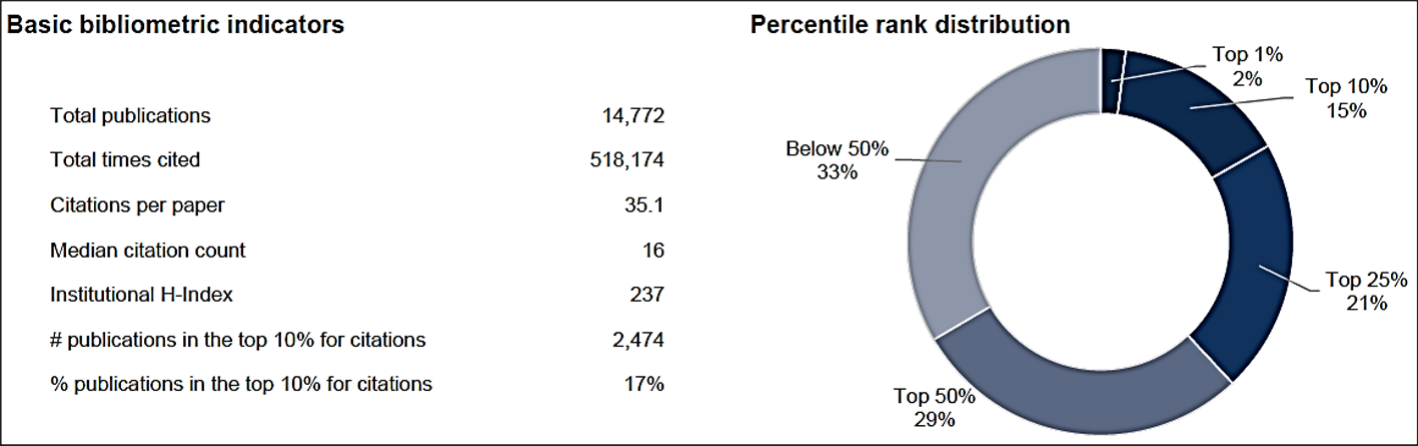
**Citation impact of FIC supported articles, 1980–2014.** The H-Index is the number of publications in a given data set that have received that same number of citations or more. The FIC Institutional H-Index of 237 indicates that FIC support was cited in 237 publications that have each been cited 237 times or more.

Publications move the knowledge base forward, strengthen leadership and institutions, and provide evidence for rational decision-making. In terms of citation impact from 1980–2014, 14,772 articles listing FIC support were cited 518,174 times with an average of 35.1 citations per paper and a median of 16 (Figure 3). Overall, 2,474 publications or 17% of all FIC-supported publications ranked in the top 10% for citations compared to all publications in Web of Science published in the same disciplines and publication years, with 2% of these publications ranked in the top 1% (Figure 3). Consequently, FIC funding results in a substantially higher proportion (i.e. double the top 1% and nearly double the top 10%) of highly cited papers than average. One reason FIC extramural programs have had such a great impact is that they support research training closely linked to international research supported by other NIH ICOs, particularly, but not exclusively, by the National Institute of Allergy and Infectious Diseases (NIAID). Several papers acknowledging support from both FIC and NIAID have been cited more than 500 times as of February 2018 [14,25,26,27,28,29,30,31,32,33].

## Conclusions

Over the decades, FIC’s programs have made significant contributions filling the pipeline of global health leaders, extending the frontiers of science and accelerating discovery. Above all, FIC invests in people—the most important resource in biomedical research—who serve on the front lines of the fight against diseases that threaten populations worldwide. FIC provides a bridge between NIH and the greater global health community by facilitating exchanges among investigators, providing training opportunities and supporting promising research initiatives in developing countries. Over the last five decades, FIC programs have provided significant research training for scientists worldwide. A high proportion of trainees, 85% to 90%, have returned to their home countries based on pre-training institutional agreements and reentry grant opportunities [34]. Health research in the 21st century is increasingly a team effort. Interdisciplinary research groups, such as those supported by FIC, have been prime movers in development of low-cost diagnostics and cost-efficient ways to prevent and treat disease.

Despite a modest budget, FIC has had a profound impact on global health during its first 50 years; from convening international conferences that stimulated the global campaign to eliminate polio, creation of the AITRP program which played a pivotal role in efforts to combat the HIV/AIDS pandemic and strengthening research capacity at institutions in LMICs responding to a wide range of global health threats. These contributions have been well-recognized by the scientific community [1,35,36].

Support of global health research and research training should not be considered charity. While LMICs benefit from these investments, high-income countries benefit as well. Examples include new approaches to prevent and treat HIV (i.e. treatment as prevention, circumcision, pre-exposure prophylazis or PREP, microbicides and combination therapies). Elimination of HIV in high-income countries depends on the above research conducted in LMICs, facilitated greatly by trainees supported through FIC programs, including research to test future HIV vaccines. Other benefits to high-income countries from global health research include development of vaccines against influenza and hepatitis B, improved treatments for breast cancer particularly for African American women, combination therapies against cancer (first demonstrated for Burkitt Lymphoma in Uganda), oral rehydration therapy, and new therapies against tuberculosis. Ongoing research in Colombia involving families genetically predisposed to an early onset form of Alzheimer’s may result in future therapies for the disease. The growth of research sites against communicable diseases exists because of collaborations between institutions and individual scientists in LMICs and high-income countries that evolved through long-term support from FIC.

## Challenges and Future Directions

While much has been accomplished, many challenges remain. These include how to build in-country support for superbly trained FIC alumni and advancing careers of U.S. investigators trained through FIC programs. These challenges include how to strengthen and sustain the viability of LMIC research institutions supported by FIC and other global health funders to respond not only to communicable diseases but to the growing burden of non-communicable diseases in LMICs. This includes how to link the results of promising research to implementation science so that future benefits can be achieved through health systems in LMICs. Responding to these challenges requires mobilizing resources from LMIC governments to share the future costs of maintaining these investments long-term. FIC is pursuing strategies to increase local government support for these efforts such as through fellowships for early career LMIC scientists linked to NIH research support in-country. Career development awards for foreign scientists and junior faculty development awarded to LMIC institutions also help to achieve these goals.

Research groups are best suited to address global health issues when teams are multinational and sensitive to local culture and context. Building such teams at institutions around the world has been and will continue to be an important FIC strategy.

FIC intends to build on this solid record of achievement during its first 50 years to prepare for future global health challenges through catalytic and cost-effective programs and advancing the new field of implementation science. FIC will continue its strong commitment to address threats from communicable diseases such as HIV/AIDS, TB, malaria and influenza.

Simultaneously, FIC will increase its commitment to address the growing burden of chronic non-communicable diseases in LMICs, including but not limited to cancer, cardiovascular diseases, child health, lung diseases, diabetes, brain disorders and mental health among others. One strategy is to link current programs to additional U.S. Centers of Excellence in the area of NCDs. FIC intends to build on existing mutually beneficial individual and institutional partnerships (both north-to-south and south-to-south) as well as forge new ones to address future global health threats utilizing shared resources with LMIC governments, foundations and other major funders of global health research.

A high priority for FIC is nurturing the next generation of global health scientists from the U.S. and LMICs through expanding training pipelines and supporting early career investigators. These goals can be advanced by building networks and continuing to link FIC training and research investments with research investments by other NIH ICOs and global health funders. One of the greatest opportunities is to strengthen participation in the Global Alliance for Chronic Diseases (GACD) and build upon the NIH-managed, FIC-coordinated World RePORT database, an online interactive map of research investments by international funding agencies, for the benefit of the global health community [37,38]. As LMICs improve their economic status, they will be better able to increase their investments in biomedical research and capacity building and with Fogarty programs, help detect and contain future infectious disease outbreaks, particularly in Africa [39].

One of the keys to future success is mentoring the next generation of LMIC and U.S. scientists. Mentoring is a critical component of all FIC research and research training programs, such as the Global Fellows and Scholars Program. A recent example of mentorship is the Clayton-Dedonder mentorship fellowship, offered as a supplement opportunity under the Global Fellows and Scholars Program using funds from the World AIDS Foundation provided to the NIH [40].

As FIC consults with its partners to plan priorities for the next 50 years, the words of its namesake, Congressman John E. Fogarty, continue to guide the way: “The nations of the world can and must share their knowledge and other resources so that people everywhere may have the blessing of better health, and through health, may move forward to new levels of peaceful productivity.”

## Data Accessibility Statement

All authors had access to the data and a role in writing the manuscript.
